# CaM/CaMKII mediates activation and proliferation of hepatic stellate cells regulated by ASIC1a

**DOI:** 10.3389/fphar.2022.996667

**Published:** 2022-12-15

**Authors:** Hui Liu, Wei-Li Lu, Hai-Qin Hong, Meng-Jun Li, Man-Ping Ye, Qiu-Fan Rao, Jin-Ling Kong, Shao-Hua Luan, Yan Huang, Qing-Hua Hu, Fan-Rong Wu

**Affiliations:** ^1^ Institute for Liver Diseases of Anhui Medical University, Hefei, China; ^2^ The Key Laboratory of Anti-inflammatory and Immune Medicines, Ministry of Education, Hefei, China; ^3^ Inflammation and Immune Mediated Diseases Laboratory of Anhui Province, School of Pharmacy, Anhui Institute of Innovative Drugs, Anhui Medical University, Hefei, China; ^4^ State Key Laboratory of Natural Medicines, Key Laboratory of Drug Metabolism and Pharmacokinetics, China Pharmaceutical University, Nanjing, China

**Keywords:** CaM, CaMKII, ASIC1a, hepatic stellate cell, cell proliferation

## Abstract

The activation of hepatic stellate cells (HSCs) is closely related to hepatic fibrosis and plays a key role in its occurrence and development. In the damaged liver, inhibition of the activation, proliferation, and clearance of HSCs is an important therapeutic strategy. However, the mechanism underlying the activation of HSCs is not completely clear. Acid-sensitive ion channel 1a (ASIC1a) is a cation channel activated by extracellular acid, which is responsible for the transport of Ca^2+^ and Na^+^ and participates in the activation of HSCs and the occurrence and development of many inflammatory diseases, suggesting that ASIC1a plays an important role in liver fibrosis. A previous study by the project team found that when the membrane channel protein ASIC1a was opened, intracellular Ca^2+^ levels increased, the expression of CaM/CaMKII in HSCs was high, and HSC was activated and proliferated. Therefore, we established an SD rat model of hepatic fibrosis and induced HSC-T6 activation by stimulating ASIC1a with acid in vitro. In vivo, CCl_4_ was used to induce liver fibrosis in rats, and different doses of KN93 (0.5, 1, and 2 mg/kg/d) and colchicine (0.1 mg/kg/d) were administered. Eight weeks later, the activities of ALT and AST in serum were measured and hematoxylin-eosin and Masson staining in liver tissue, and immunohistochemistry analysis were performed in SD rats. The expressions of ASIC1a, α-SMA, Collagen-1, CaM, and CaMKII were detected. In vitro, we activated HSC-T6 cells by stimulating ASIC1a with acid. The results showed that inhibition of ASIC1a could improve acid-induced HSCs activation. In addition, CaM/CaMKII was expressed in HSC of rats with hepatic fibrosis regulated by ASIC1a. After blocking or silencing the expression of CaMKII, the fibrosis marker protein can be down-regulated. KN93 also reduced inflammation and improved the activation, proliferation and fibrosis of HSC. In summary, we concluded that CaM/CaMKII participates in ASIC1a regulation of the proliferation and activation of HSC and promotes the occurrence of liver fibrosis.

## Introduction

Hepatic fibrosis (HF) is a common pathological process of chronic liver disease caused by various factors and an intermediate stage of liver cirrhosis. There are many causes of liver fibrosis, including viruses, alcohol, and chemical drugs. Chronic hepatitis is the main cause of liver fibrosis in China. Among them, 25% of 40% of liver fibrosis eventually develops into liver cirrhosis, leading to liver cancer, which seriously harms human health and places a huge burden on society (Caligiuri et al., 2021). Studies have shown that HF is a programmed response of the body to injury, and it is a dynamic and reversible lesion. Its formation and development involve many factors, among which the activation and proliferation of hepatic stellate cells (HSCs) are the core links in the formation of hepatic fibrosis (Liu et al., 2019). When the liver is damaged, HSC activates and proliferates, transforms into muscle-like fibroblasts, expresses α-smooth muscle actin (*α*-SMA), and increases the synthesis and secretion of extracellular matrix (ECM), which leads to the imbalance between ECM synthesis and degradation, resulting in excessive ECM deposition in liver tissue, forming “fiber scar” (Ezhilarasan et al., 2018; Zhang et al., 2019; El-Maadawy et al., 2020; Martí-Rodrigo et al., 2020). Therefore, inhibiting the activation and proliferation of HSC is key to controlling the progression of liver fibrosis.

Acid substances can be produced during body metabolism, and tissue acidification is a common phenomenon under physiological and pathological conditions. The chronic pathological process of liver fiber leads to chronic inflammation and injury of the liver for a long time, and inflammation is often accompanied by tissue acidification. pH in inflammation, ischemia, and tumors can be reduced to less than 6.0 (Harmon et al., 2019; Tcymbarevich et al., 2019). Acid-sensitive ion channels (ASICs) are transmembrane cation channels activated by H^+^ after extracellular acidification. This channel allows Na^+^ and Ca^2+^ to flow into the cell, causing a series of physiological and pathological changes (Sun et al., 2017). ASICs are widely distributed *in vivo*; however, there are differences in the distribution of different subunits. Among them, ASIC1a has become a research hotspot because of its role in mediating Ca^2+^ influx, extensive biological functions, and its important pathological significance. It has been found that ASIC1a participates in the occurrence and development of many diseases, such as synovitis, liver cancer, and cardiovascular diseases, which lead to local hypoxia, anaerobic glycolysis of tissues, accumulation of hydrolytic protons of lactic acid and ATP, decrease in pH value around tissues, opening of ASIC1a channels, inflow of Ca^2+^ into cells, and participation in metabolic processes under various cellular and histopathological conditions (Rehni et al., 2019; Zhang et al., 2020). In a previous study by our group, we found that there was an expression of ASIC1a in rat liver and HSC, and the expression of ASIC1a increased in rats with HF and HSCs stimulated by Platelet-derived growth factor (PDGF). Inhibition of ASIC1a activity significantly reduced the levels of inflammatory factors such as IL-1 and IL-6 in the serum of rats with HF, decreased the protein expression of *α*-SMA, transformating growth factor betal (TGF-β1), nuclear factor kappa B(NF-κB), and collagen-I in liver tissue, and reduced the degree of HF. Further studies have found that ASIC1a participates in the functional and metabolic processes of HSC. When stimulated by acid or PDGF, ASIC1a is activated and channels are opened, which increases Ca^2+^ influx into HSC, promotes the proliferation and activation of HSC, and regulates collagen secretion through matrix metalloproteinases-13 (MMP-13) and tissue inhibitor of metalloproteinases-1 (TIMP-1) (Pan et al., 2014; Kong et al., 2021; Luan et al., 2022). However, this study only observed that ASIC1a is involved in the process of HF due to the opening of the ASIC1a channel, which is related to the regulation of HSC function. Changes in the intracellular microenvironment of HSC and the intracellular mechanisms affecting the activation and proliferation of HSCs are still unclear.

As a ubiquitous and important second messenger substance, Ca^2+^ is widely involved in important pathophysiological processes in cells, and its small changes can affect the normal physiological function of cells. Ca^2+^ influx is a key link in the signal transduction process of activation of hepatic stellate cells (Li et al., 2020; Wang et al., 2021a). Calmodulin (CaM) is widely expressed in various cells. It is the intracellular receptor of Ca^2+^, which can bind to Ca^2+^ and has a variety of functions. It is inactive and calcium-dependent. When the concentration of intracellular Ca^2+^ increases, calmodulin stimulates, binds to intracellular Ca^2+^, and produces a Ca^2+^-CaM complex. Simultaneously, CaM configuration changes, exposing hydrophobic regions, binding to calmodulin-dependent target enzymes, activating target enzymes, producing a series of biological changes and reactions, and transmitting signals downstream (Park et al., 2019; Akizuki et al., 2021).

The downstream target enzymes regulated by the Ca^2+^-CaM complex include phosphorylase kinase, guanosine cyclase, phospholipase A2, myosin light chain kinase, phosphodiesterase, and calmodulin kinase. Calmodulin-dependent protein kinases (CaMK) are serine/threonine protein kinases that are mainly divided into two categories: specific kinases and multifunctional kinases (Takemoto-Kimura et al., 2017). CaMKII is a multifunctional kinase (Gaitán-González et al., 2021). As the most important downstream regulatory protein in the Ca^2+^ signaling system, CaMKII is widely involved in many physiological processes, such as cell proliferation, apoptosis, cycle regulation, and neurotransmission cell secretion. Zhao et al. (2018) found that CaMKⅡ has an important physiological regulatory function in human umbilical vein endothelial cells. After inhibition of its activity, endothelial cell proliferation was significantly reduced. Transient receptor potential channel 4 (trpv4) regulates the proliferation of oral squamous cell carcinoma (OSCC). Ca^2+^ enters cancer cells, activates CaMKII, regulates downstream AKT phosphorylation, and promotes the growth of OSCC cells (Fujii et al., 2020). However, there are few reports on CaMKII-mediated regulation of HSC function in HF.

On the basis of observing the effect of ASIC1a on the activation and proliferation of HSC, we started with the intracellular signal CaM/CaMKII of HSC to find a new target for reversing liver fibrosis. Among the events included in the molecular mechanism of the HSC pathological process by ASIC1a, we determined the internal relationship and essence of various factors of Ca^2+^ and CaM/CaMKII and clarified the cellular and molecular mechanisms of HSC activation and proliferation to provide a new strategy for further research on anti-fibrosis drugs.

## Materials and methods

### Antibodies and reagents

ASIC1a (1:1,000 dilution, Bioss), Collagen-1 (1:1,000 dilution, Proteintech), *α*-SMA (1:1,000 dilution, Bioss), *β*-Actin (1:1,000 dilution, Proteintech), CaM (1:1,000 dilution, Affinity), CaMK II (1:1,000 dilution, Affinity), NFAT (1:1,000 dilution, Affinity), MMP-13 (1:1,000 dilution, Bimake.cn), NF-κB (1:1,000 dilution, Bimake.cn), KN93, and colchicine were purchased from MedChemExpress (MCE, United States). Spider-venompeptide (PcTx-1) was purchased from Abcam (Cambridge, United States). High-sugar DMEM culture medium and phosphate buffered saline (PBS) were purchased from HyClone (United States). Horseradish enzyme labeled anti-rabbit IgG and horseradish enzyme labeled anti-mouse IgG were purchased from Bioss (Beijing, China). PVDF membrane was purchased from Millipore (United States). Fluo-3AM calcium fluorescent probe was purchased from beyotime (Shanghai, China).Lipofectamine 2000, TRIzol Reagent, and Opti-MEM were purchased from Invitrogen (Invitrogen, United States). Amiloride was purchased from Sigma-Aldrich (St. Louis, MO, United States). Rapid Gel Preparation Kits were purchased from EpiZyme (EpiZyme, China). Cell cycle detection kit and CCK-8 cell proliferation toxicity assay kit were purchased from BestBio (Beijing, China).

### Cell culture and treatment

The rat hepatic stellate cell line HSC-T6 was provided by Procell Life Technology Co. Ltd. The cells were cultured in a high-glucose DMEM, which was improved by adding 20% fetal bovine serum and 1% penicillin-streptomycin solution, and the medium was changed every 2 days. HSC-T6 cells were planted according to the 5 × 10^5^ power cell density in each well, and the pH value was adjusted to 6.0 with .01 mol/ml HCL in the culture medium. Finally, the cells were incubated at 37°C in a 5% CO_2_ cell incubator for 24 h, and the fine cells were collected for detection.

### Cell transfection

ASIC1a-siRNA, negative control, and pcDNA3.1 vectors carrying ASIC1a were provided by Hanheng Biotechnology (Shanghai) Co., Ltd. HSC was inoculated in a 6-well plate with 5 × 10^5^ cells per well, and continued to be cultured in the incubator until adherent. After 24 h of culture, According to the operating rules of the manufacturer, ASIC1a-siRNA, OE-ASIC1a and negative control targeting ASIC1a were introduced into the cells with Lipofectamine 2000 at the concentration of 50 nM and Opti-MEM, respectively. Normal medium was changed after 4 h of transfection. The sequence targeting ASIC1a-siRNA was as follows: sense, 5-GCG​UGA​AUC​UAC​GAC​AGA​TT-3; antisense, 5-UCU​GUC​GUA​GAA​UCA​CGC​TT-3′. The siRNA sequence of the negative control was 5-UUC​UCC​GAA​CGU​GUC​ACG​UTT-3, and the antisense sequence was 5-ACG​UGA​CAC​GGA​ATT-3′. CaMKII-siRNA, negative control, and pcDNA3.1 vector carrying CaMKII were all provided by Generalbiol (Generalbiol, China). The sequence of CaMKII-siRNA was as follows: sense: 5-GGG​UAA​AGA​UAA​ACA​ACA​ATT-3; Antonym: 5-UUG​UUG​UUU​AUC​UUU​ACC​CTT-3; the siRNA sequence of the negative control was sense: 5-UUC​UCC​GAA​CGU​GUC​ACG​UTT-3, antisense: 5-ACG​UGA​CAC​GUU​CGG​AGA​ATT-3′. Transfection efficiency was detected by western blotting and qRT-PCR.

### Analysis of cell survival rate and proliferation

The cell survival and proliferation rates were measured using CCK-8 colorimetry. The HSCs were inoculated with 5 × 10^5^ cells per well in a 96-well plate and cultured in the incubator until they adhered to the wall. After 24 h of culture, the medium containing a lower percentage of serum (1% fetal bovine serum) was starved for 24 h. For the viability assay, The culture medium with different concentrations of PcTx-1 (50, 100, 150, 200, 400, and 800 ng/ml) continued to incubate for 24 h. For the proliferation assay, Fine cells were pretreated with different concentrations of KN93 (1.25, 2.5, 5, 10, 20, and 40 mmol/L) for 24 h. HSCs were stimulated with pH 6.0 HCl and different concentrations of KN93 for 24 h. After treatment, the CCK-8 solution 10 uL was added to each well and incubated for 2–4 h. A multimode reader (BioTek, Winooski, VT, United States) was used to measure the absorbance of the plate at 450 nm.

### Cell cycle analysis

The HSC-T6 cell cycle was analyzed using a cell cycle detection kit (Beibo, China). First, well-growing HSCs were inoculated into a 6-well plate. The cells were then treated with different stimuli. Briefly, the cells in each group were digested with trypsin, cold PBS was prepared in advance, pay attention to gently blowing and mixing to prevent cells from forming clumps, and the obtained HSC-T6 cells were suspended in 75% cold ethanol at 4°C overnight or −20°C for 1 h. The fixed cells were washed twice with PBS, ribonuclease A (RNaseA) 20 μl was added, and the cells were incubated for 30 min at 37°C. Then, 400 mesh cells were filtered, and 400 μl propidium iodide staining buffer was added and incubated at 4°C for 30 min. After staining, the cells were analyzed using flow cytometry (BD FACS Verse, United States). The excitation wavelength was 488 nm. The proportion of cells in each cycle was analyzed using the ModFit software (BD Biosciences, United States).

### Detection of intracellular [Ca^2+^]i by laser scanning confocal microscope

Determination of [Ca^2+^]i in HSC under extracellular acidification by Fura-3/AM probe and laser confocal microscope: HSC were inoculated in a Petri dish with 5 × 10^5^ cells per well, rinsed twice with Hanks solution, 150 μl mixed working solution (5 mmol/ml Fluo-3/AM, .02%F-127) of Fluo-3/AM and F-127 was added to the Petri dish, incubated at 37°C for 30 min, and washed three times again to remove excess dyes. A small Hanks’ solution was retained to balance the cell for 10 min, and the intracellular Ca^2+^ concentration was detected in 10 min. When the intracellular Ca^2+^ concentration was measured in the presence of calcium, Hanks’ solution was replaced with D-Hanks’ solution. The cells were dynamically scanned under a laser confocal microscope for 20 s, and extracellular solution (adjusted to pH 6.0) was injected into the cover slide with a microsampler. Eight cells were taken from nine cells under a microscope, and changes in intracellular calcium fluorescence intensity were dynamically observed.

### Immunofluorescence

HSC-T6 cells (5 × 10^5^) were seeded in a 6-well plate and cultured for 24 h after adhering to the wall with the corresponding concentration of stimulation. The treated cells were removed from the culture medium and washed with PBS 3 times, each time 5 min. After 10 min, the cells were fixed in 4% paraformaldehyde, sealed with PBS containing 10% bovine serum albumin (BSA) for 20 min, and incubated overnight with a mixed primary antibody of CaM/CaMKII and *α*-SMA (1:100 dilution) at 4°C. After PBS washing for three times, FITC-labeled anti-mouse IgG and Alexa Fluor/594-labeled anti-rabbit were incubated for 1 h at room temperature in the dark. After washing with PBS, nuclei were stained with 4,6-diimino-2-phenylindole (DAPI), and then the images were taken by laser confocal photography.

### Western blot analysis

HSC-T6 cells were evenly mixed with PMSF and RIPA cell lysates at a ratio of 1:100, and the total protein was extracted by lytic cells on ice. The protein concentration was measured using a BCA Protein Quantitative Kit (Beyotime, Shanghai, China). Equal amounts of protein were separated by 10% or 12% sodium dodecyl sulfate-polyacrylamide gel electrophoresis (SDS-PAGE) and transferred to polyvinylidene fluoride (PVDF) membranes (Millipore, Bedford, MA, United States). The membrane was sealed in TBS/Tween 20 for 2 h with 5% milk and then incubated with the primary antibody overnight at 4°C. Then, the membrane was washed with TBST three times and incubated with horseradish peroxidase (HRP)-coupled secondary antibody (1:10,000). ECL chemiluminescence (Advansta, CA, United States) was used to analyze the expression of proteins in imprinting on gel imaging equipment (ChemiDoc MP imaging system (Bio-Rad, United States). The protein band strength was quantified by ImageJ software, and the relative expression of the target protein was calculated with *β*-Actin as the standard.

### Real-time quantitative polymerase chain reaction (RT-PCR)

Total RNA from HSC-T6 cells was extracted using a TRIZOL reagent. An appropriate amount of (10 μl) wa s added to the obtained RNA, dissolved, and quantified with Nanodrop 2000 (Thermo Scientific, United States). The PrimeSciptTM RT kit (Takara) was used according to the manufacturer’s instructions to extract cDNA from purified RNA. cDNA analysis was performed on a Bio-Rad CFX-linkedfluorescence quantitative PCR system using SYBR PreMix Ex TaqII (TAKARA), with a total of 96 wells. All PCR amplification results were obtained in triplicate, and the average values were recorded. The gene expression level, polymerase chain reaction efficiency, and standard deviation were analyzed, and the efficiency was close to 100%. The expression of relevant mRNA was calculated using the 2^−△△Ct^ method. *β*-Actin was used as the standard for normalization. Several gene primer sequences used for real-time PCR are shown in Table 1.

**TABLE 1:** Primer sequences of several genes for real-time PCR.

Gene	Forward (5′ > 3′)	Reverse (5′ > 3′)
*β*-Actin	5′–GAG​CGC​AAG​TAC​TCT​GTG​TG–3′	5′–CCT​GCT​TGC​TGA​TCC​ACA​TC–3′
ASIC1a	5′–CGG​CTG​AAG​ACC​ATG​AAA​GG–3	5′–AAG​GAT​GTC​TCG​TCG​GTC​TC–3′
*α*-SMA	5′–GAG​GGA​TCC​TGA​CCC​TGA​AG–3′	5′–CCA​CGC​GAA​GCT​CGT​TAT​AG–3′
Collagen-I	5′–ACC​TCA​GGG​TAT​TGC​TGG​AC–3′	5′–GAC​CAG​GGA​AGC​CTC​TTT​CT–3′
CaMK II	5′- AGC​CAT​CCT​CAC​CAC​TAT​GCT​G-3′	5′-GTG​TCT​TCG​TCC​TCA​ATG​GTG*G*-3′
CaM	5′- GAA​CCC​AAC​AGA​GGC​TGA​ACT-3′	5′-CAC​GGA​TTT​CTT​CTT​CGC​TAT​C-3′
MMP-13	5′-GCA​GTC​TTT​CTT​CGG​CTT​AGA​G-3'	5′-GTA​TTC​ACC​CAC​ATC​AGG​AAC​C-3′
NF-kB	5′-GTG​GCC​CAC​ATC​GGT​TAA​CT-3'	5′- CCC​TTC​AAC​TGT​CAA​CCT​CA-3′
NFAT	5′-TGC​TCC​TCC​TCC​TGC​TGC​TC-3'	5′-CGT​CTT​CCA​CCT​CCA​CGT​CG-3′

### Establishment of experimental animals and experimental models

Sixty SPF male SD rats weighing 180–200 g were selected. Experimental rats were provided by the Experimental Animal Center of Anhui Medical University and raised in the animal room (general breeding room) pharmacy of the school. After 1 week of adaptation, the rats were randomly divided into six groups: normal control group, model group, KN93 low (.5 mg/kg), medium (1 mg/kg) and high (2 mg/kg) dose groups, and positive drug colchicine group, with 10 rats in each group. Except for the normal control group, all other groups were intraperitoneally injected with 40% CCl_4_ .1 ml/100 g twice a week for 12 weeks to establish a model of HF in CCl_4_ rats. From the fourth week of modeling, each administration group was injected with different doses of KN93 every day, and the normal and model groups were injected with the same amount of NS through the tail vein for 8 weeks. After 8 weeks of modeling, all rats were fasted for 8 h, weighed, anesthetized, blood was collected from the abdominal aorta, serum was routinely prepared, the liver was taken, stored at −80°C, and tested for related indexes.

### Pathomorphological examination of rat liver

The liver lobes of the rats in each group were fixed with 4% neutral formaldehyde, and routine paraffin sections were prepared for pathological observation. Hematoxylin-eosin (HE) staining and Masson collagen fiber staining were used to observe the pathological changes in the liver under a light microscope. Briefly, tissue sections were dewaxed with xylene, washed with different concentrations of ethanol, stained with hematoxylin dye for 3–5 min, and differentiated with differentiation solution after washing. The tissue slices were dehydrated in 85% and 95% gradient alcohol for 5 min and stained with eosin. The slices were then washed with different concentrations of ethanol and dehydrated routinely to make them transparent and neutral gum sealed. The tissue sections were dewaxed with xylene for Masson staining, washed with different concentrations of ethanol, soaked overnight in MassonA solution, and then stained with 1% hydrochloric acid alcohol for 1min with a mixture of MassonB and MassonC solutions. After washing, the sections were stained with MassonD solution for 6 min, washed, and stained with MassonE solution for 1 min. They were not washed but drained slightly and dyed directly into the MassonF solution for 2–30 s. The slices were then rinsed with 1% glacial acetic acid and dehydrated with anhydrous ethanol. The slices were sealed with anhydrous ethanol for 5 min and xylene for 5 min to make them transparent and neutral gum. After scanning on the PANNORAMIC digital section scanner of 3DISTECH, the degree of inflammation and fibrosis of the liver tissue in each group was observed using CaseViewer software.

### Detection of liver function and serological indexes in rats with hepatic fibrosis

Blood was collected, and the serum was separated. The liver function indices of ALT and AST were detected using the ultraviolet-lactate dehydrogenase and malate dehydrogenase methods, respectively. A Hitachi-3100 automatic biochemical analyzer was used to detect the activity levels of ALT and AST in rat serum according to the manufacturer’s instructions.

### Immunohistochemistry

The wax blocks of liver tissue were sectioned, dewaxed, and repaired routinely. 3% BSA was dripped into the tissue circle to cover the tissue evenly and, for 30 min, was sealed at room temperature. The samples were then incubated with anti-*α*-SMA antibody (1:200), and the slices were placed flat in a wet box at 4°C for overnight incubation. The membranes were then incubated with secondary antibodies coupled with anti-rabbit or anti-mouse horseradish peroxidase (HRP). After washing with PBS and incubating with diaminobenzidine (DAB) solution, the nuclei stained with hematoxylin were blue, and the positive expression of DAB was brown. Immunostained samples were observed using a slide scanner (3DHISTECH, Budapest, Hungary).

### Statistical analysis

GraphPadPrism7 software was used to process the data and generate the charts. Single factor analysis of variance (One-way ANOVA) was used to compare the means of multiple groups of samples, and a *t*-test analysis of a completely random design was used to analyze the statistical differences between the two groups. All data were repeated three times and expressed as the mean ± standard deviation, *p* < .05, and the difference was considered statistically significant.

## Results

### Inhibition of ASIC1a and improvement of acid-induced HSCs activation

**FIGURE 1:**
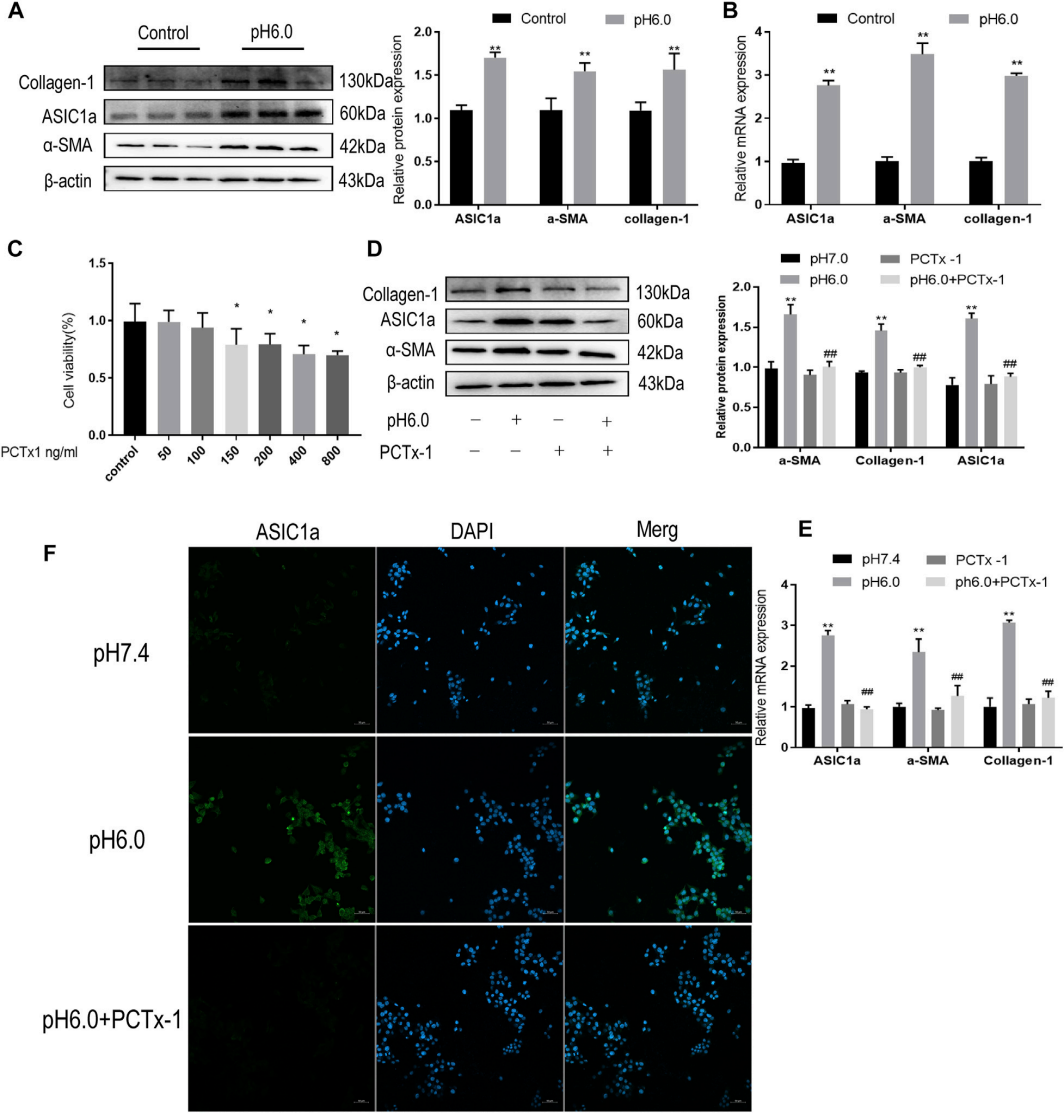
Inhibition of ASIC1a improves acid-induced activation of HSCs. **(A)** Western blotting was used to detect the expression of collagen-1, ASIC1a and *α*-SMA in HSCs stimulated by pH 6.0. **(B)** qRT-PCR was used to detect the mRNA levels of collagen-1, ASIC1a, and *α*-SMA in HSCs stimulated by pH 6.0. **(C)** The effect of PcTx-1 on the viability of HSCs was detected by CCK8. **(D)** Western blotting was used to detect the expression of collagen-1, *α*-SMA, and ASIC1a in HSCs under the intervention of PcTx-1. **(E)** qRT-PCR to detect the expression of collagen-1, ASIC1a, and *α*-SMA under the intervention of PcTx-1. **(F)** Immunofluorescence to detect the effect of PcTx-1, a specific blocker of ASIC1a channel, on ASIC1a in HSCs. (Scale Bar, 50 μM). Data are expressed as the mean ± SD (*n* ≥ 3). *p < .05, **p < .01 vs. Control group; ^#^ *p* < .05, ^##^ *p* < .01 vs. pH 6.0 group.

Our previous studies have shown that acid can stimulate ASIC1a to regulate the activation and proliferation of HSCs and further verify the activation of the HSC-T6 cell model induced by ASIC1a. Acidification of the HSC-T6 culture environment to pH 6.0 can open ASIC1a channels and increase the expression of HF markers Collagen-1, *α*-SMA, and ASIC1a, as well as the level of mRNA (Figures 1A, B). Simultaneously, we detected the effect of psalmooxin-1 (PcTx-1) on the viability of HSC using the CCK8 method. After blocking ASIC1a with a specific inhibitor, PcTx-1. When the concentration of PcTx-1 detected by CCK8 was 100 ng/ml, PcTx-1 had the least effect on the viability of HSC-T6 cells. (Figure 1C). Therefore, it was administered at this concentration. The levels of *α*-SMA, collagen-I, and ASIC1a protein and mRNA decreased after PcTx-1 intervention (Figures 1D, E). The expression of ASIC1a on the membrane surface of HSC-T6 cells was detected using immunofluorescence. The results showed that extracellular acid promoted the expression of ASIC1a while PcTx-1 inhibited the channel and reduce its activity (Figure 1F). These results suggest that the inhibition of ASIC1a by PcTx-1, a specific blocker, can improve the acid-induced proliferation and activation of HSCs.

### CaM/CaMKⅡ is expressed in HSC of rats with HF regulated by ASIC1a

**FIGURE 2:**
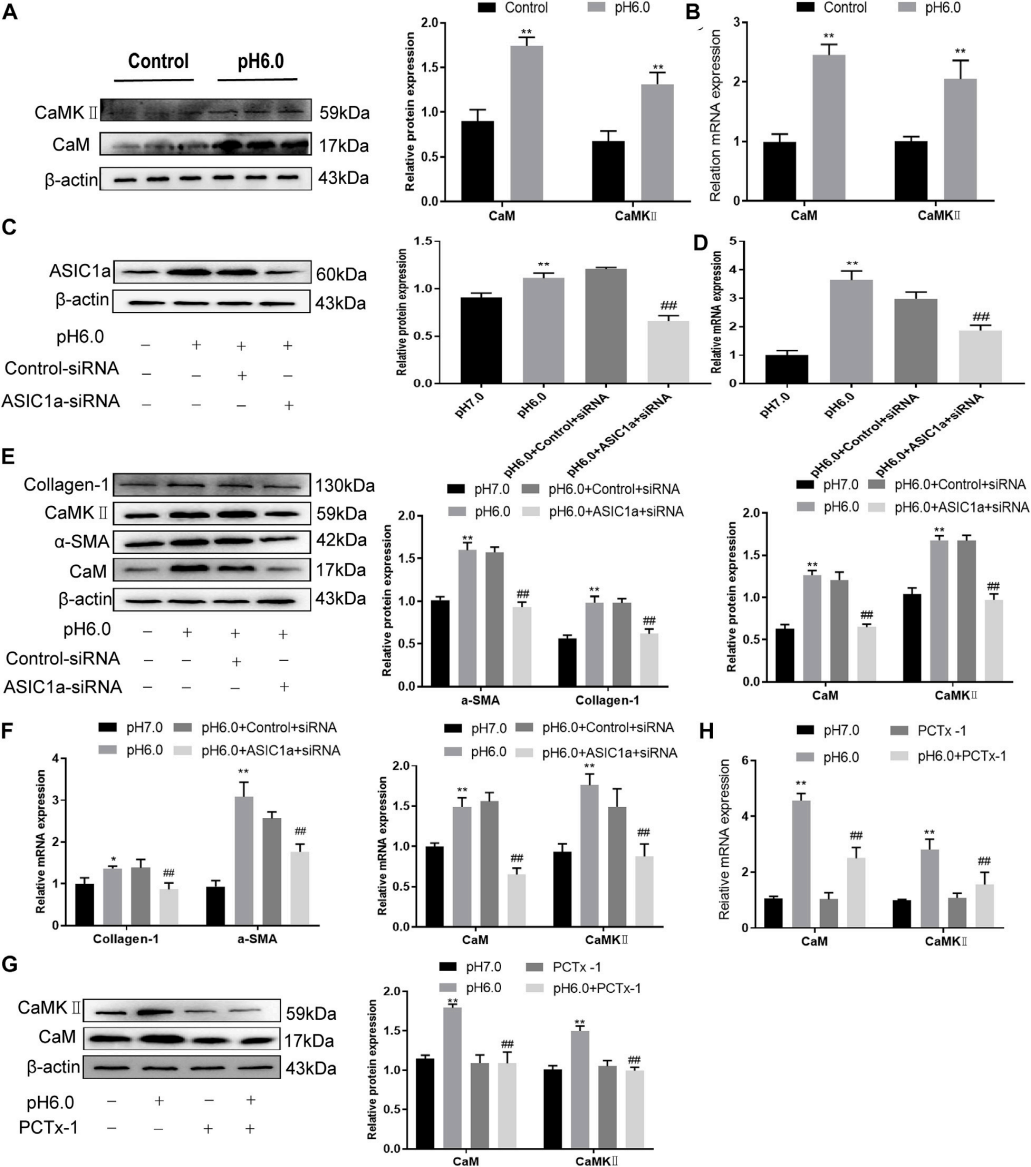
Effect of ASIC1a-siRNA transfection and PcTx-1 on ASIC1a, *α*-SMA, and collagen-Ι as well as CaM and CaMKⅡ expression in HSC-T6 cells. **(A)** Western blotting was used to detect the expression of CaM and CaMKⅡ protein in HSCs stimulated by pH 6.0. **(B)** The mRNA levels of CaM and CaMKⅡ in HSCs stimulated by pH 6.0 were detected by qRT-PCR. **(C)** ASIC1a protein level in HSCs was detected by Western blotting after silencing ASIC1a. **(D)** ASIC1a mRNA level was detected by qRT-PCR after silencing ASIC1a. **(E)** Expression of CaM, CaMKII and fibrosis protein in HSCs after silencing ASIC1a. **(F)** mRNA level of CaM, CaMKII, and fibroprotein in HSCs after silencing ASIC1a. **(G)** CaM and CaMKⅡ protein levels in HSCs treated with PcTx-1 were detected by Western blotting. **(H)** The mRNA levels of CaM and CaMKⅡ in HSCs treated with PcTx-1 were detected by qRT-PCR. Statistical analyses were performed using *t*-test. Data are expressed as the mean ± SD (*n* ≥ 3). *p < .05, **p < .01 vs. Control group; ^#^ *p* < .05, ^##^ *p* < .01 vs. pH 6.0 group.

**FIGURE 3:**
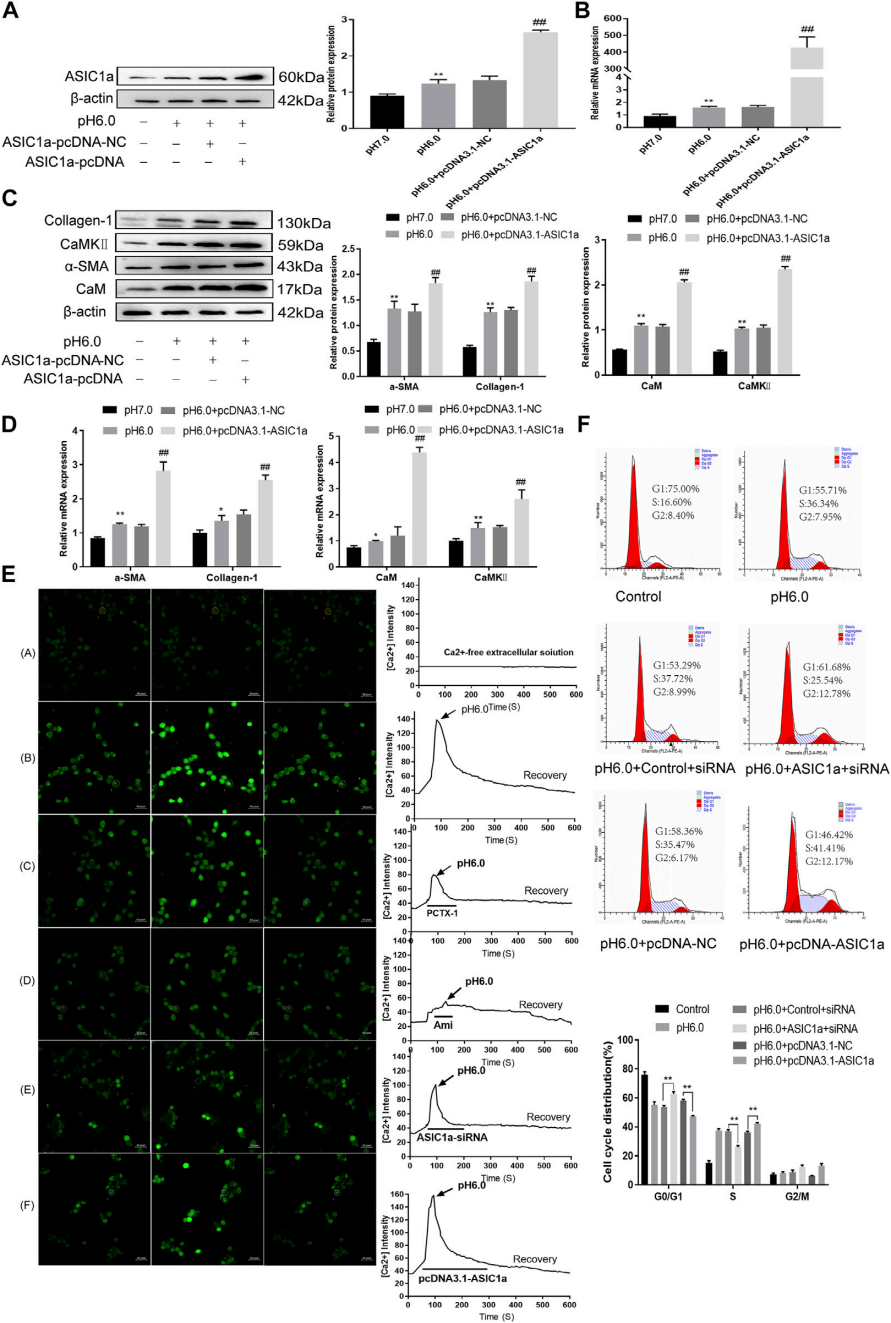
The expression of CaM/CaMKⅡ was detected in HSC-T6 cells regulated by ASIC1a. **(A)** The level of ASIC1a protein in HSCs was detected by Western blotting after overexpression of ASIC1a. **(B)** The mRNA level of ASIC1a in HSCs was detected by qRT-PCR after overexpression of ASIC1a. **(C)** The expression of CaM, CaMKⅡ and fibrosis protein in HSCs was detected by Western blotting after overexpression of ASIC1a. **(D)** After overexpression of ASIC1a, qRT-PCR was used to detect the level of mRNA of CaM, Cam KII and fibrosis protein in HSCs. **(E)** After ASIC1a was silenced or overexpressed, the effect of HSCs cells cycle was detected by flow cytometry. **(F)** Laser confocal scanning (Scale Bar, 50 μm) to detect the effect of ASIC1a on Ca^2+^ concentration in HSCs. (Scale Bar, 50 µM).Data are expressed as the mean ± SD (*n* ≥ 3). *p < .05, **p < .01 vs. Control group; ^#^ *p* < .05, ^##^ *p* < .01 vs. pH 6.0 group.

When the membrane channel protein ASIC1a was opened, the intracellular Ca^2+^ levels increased, the expression of CaM/CaMKII in HSC was increased, and HSCs were activated and proliferated. ASIC1a plays an important role in this process. First, acidification of HSC-T6 to pH 6.0 resulted in the increased expression of ASIC1a and protein and mRNA levels of CaM and CaMKII, and the difference was statistically significant (*p* < .05) (Figures 2A, B). Simultaneously, we established models of drug blocking, overexpression, and silencing of ASIC1a, and the transfection efficiency was verified by western blotting and qRT-PCR. The results showed that the mRNA and protein levels of ASIC1a decreased after silencing of ASIC1a, and the difference was statistically significant (*p* < .05) (Figures 2C, D). In addition, HSCs were treated with PcTx-1, a specific inhibitor of ASIC1a, at 100 ng/ml and then adjusted to the culture medium pH 6.0 for 24 h. The results of western blotting and qRT-PCR showed that after blocking the expression of ASIC1a with the specific inhibitor PcTx-1 or downregulating the expression of ASIC1a with specific ASIC1a-siRNA, the expression of ASIC1a and HF markers, collagen-1, *α*-SMA, CaM, and CaMKII, decreased. Compared with the model group, the difference was statistically significant (Figures 2E–H), and the result of the ASIC1a overexpression plasmid group was opposite to that of the above-mentioned results (Figures 3A–D). Laser confocal microscopy was used to study the effect of ASIC1a on the concentration of Ca^2+^ in HSC-T6 cells. When the extracellular solution was D-Hanks’ buffer (without Ca^2+^), there was no change in HSC-T6 cytoplasmic fluorescence before, during, and after adding pH 6.0 acid. The FI data analysis showed that the change curve was nearly flat (Figure 3E A). When the loaded cells were placed in Hanks buffer, the cytoplasmic Ca^2+^F340/F380 ratio changed significantly before, during, and after the addition of pH 6.0 acid. After adding acid for 20 s, the cytoplasmic Ca^2+^ FI value increased sharply to a peak (fluorescence was close to saturation), the fluorescence began to weaken, and the ratio decreased slowly with time. After stopping the administration, the ratio continued to decrease, and 100 s after withdrawal, the cytoplasmic Ca^2+^ ratio did not return to the basic level before administration (Figure 3E B). The cytoplasmic Ca^2+^ FI value changed significantly (Figure 3E C, D) in amiloride 100 um and PcTx-1 100 ng/ml groups, indicating that extracellular acidification-activated ASIC1a could increase the intracellular Ca^2+^ concentration in HSC-T6 cells, and blocking ASIC1a significantly inhibited the increase in intracellular Ca^2+^ concentration in HSC-T6 cells. Silencing of ASIC1a significantly inhibited the increase in intracellular Ca^2+^ concentration in HSC-T6 cells (Figure 3E E), and the intracellular Ca^2+^ concentration in HSC-T6 cells increased significantly after the overexpression of ASIC1a (Figure 3E F). It has been suggested that ASIC1a exists in HSC-T6 cells as an acid receptor, and whether its channel is open is directly related to the concentration of Ca^2+^. In addition, flow cytometry showed that acid-induced accelerated G1/S transformation was reduced after exposure to ASIC1a-siRNA, which hindered cycle progression and inhibited the proliferation of HSC-T6 cells, while the overexpression of ASIC1a significantly increased the S phase and accelerated the G1/S transition of HSC-T6 cells, thus aggravating the proliferation of activated HSC-T6 cells (Figure 3F). The above data suggest that acid-stimulated ASIC1a can promote the activation of HSC and CaM/CaMKII is expressed in HSCs of rats with HF regulated by ASIC1a.

### Effect of CaM/CaMKⅡ on activation and proliferation of HSC

**FIGURE 4:**
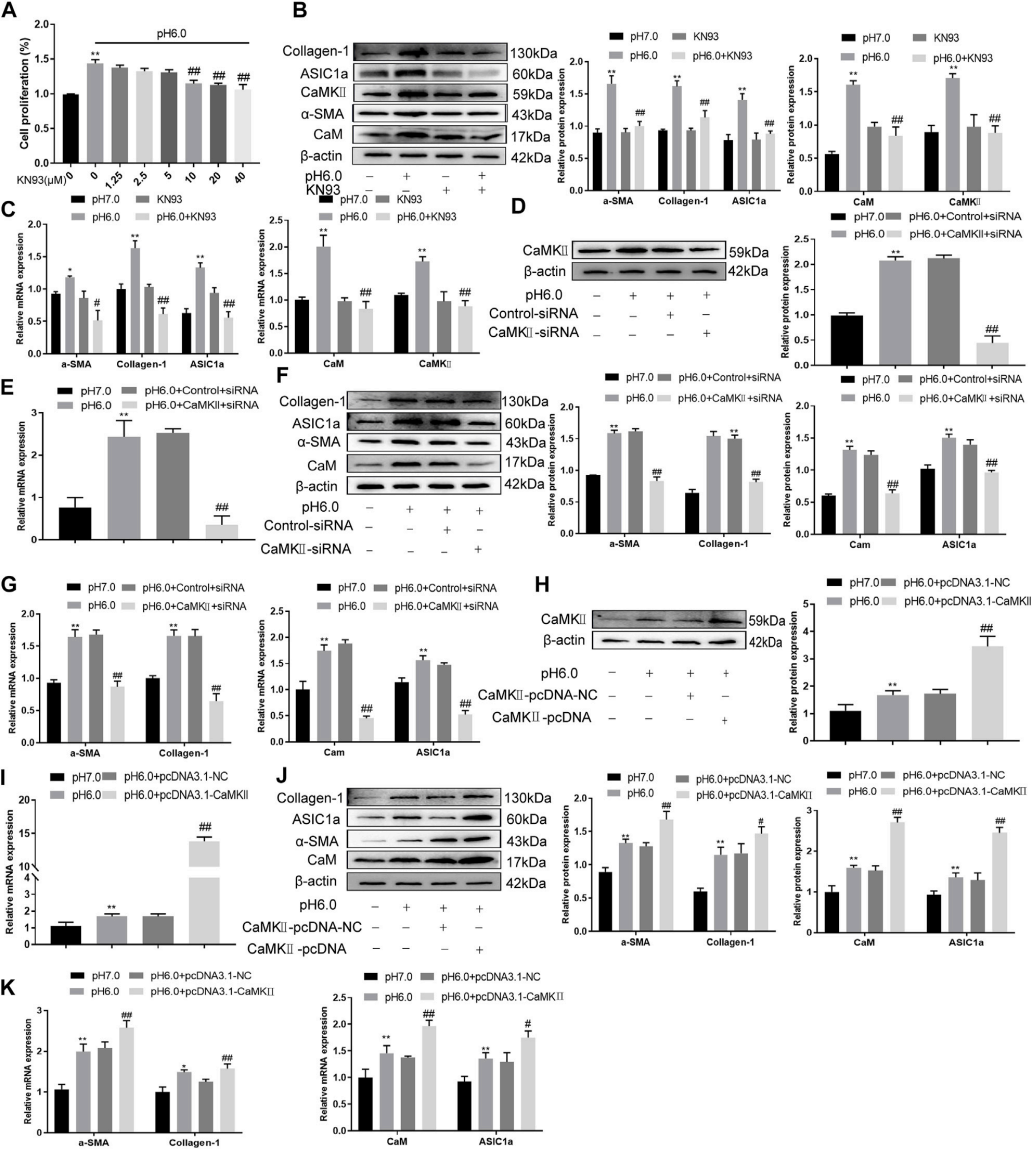
KN93 can inhibit the expression of CaM/CaMKⅡ in HSC-T6. **(A)** CCK-8 method was used to detect the effect of KN93, a specific inhibitor of CaMKⅡ, on acid-induced hematopoietic stem cell proliferation. **(B)** Western blotting was used to detect the levels of fibroproteins, CaM, and CaMKⅡ in HSCs treated with KN93. **(C)** qRT-PCR was used to detect the mRNA levels of fibroproteins, CaM and CaMKⅡ in HSCs treated with KN93. **(D)** After CaMKⅡ was silenced, CaMKⅡ protein levels in HSCs were detected by Western blotting. **(E)** After silencing CaMKⅡ, the mRNA level of CaMKⅡ was detected by qRT-PCR. **(F)** After silencing CaMKⅡ, the levels of CaM and fibrosis protein in HSCs. **(G)** mRNA levels of CaM and fibroproteins in HSCs after silencing CaMKⅡ. **(H)** The level of CaMKⅡ protein in HSCs was detected by Western blotting after overexpression of CaMKⅡ. **(I)** The mRNA level of CaMKⅡ in HSCs was detected after overexpression of CaMKⅡ. **(J)** Detection of CaM and fibrosis protein expression in HSCs after overexpression of CaMKⅡ. **(K)** mRNA levels of CaM and fibroproteins in HSCs after overexpression of CaMKⅡ. Data are expressed as the mean ± SD (*n* ≥ 3). *p < .05, **p < .01 vs. Control group; ^#^ *p* < .05, ^##^ *p* < .01 vs. pH 6.0 group.

**FIGURE 5:**
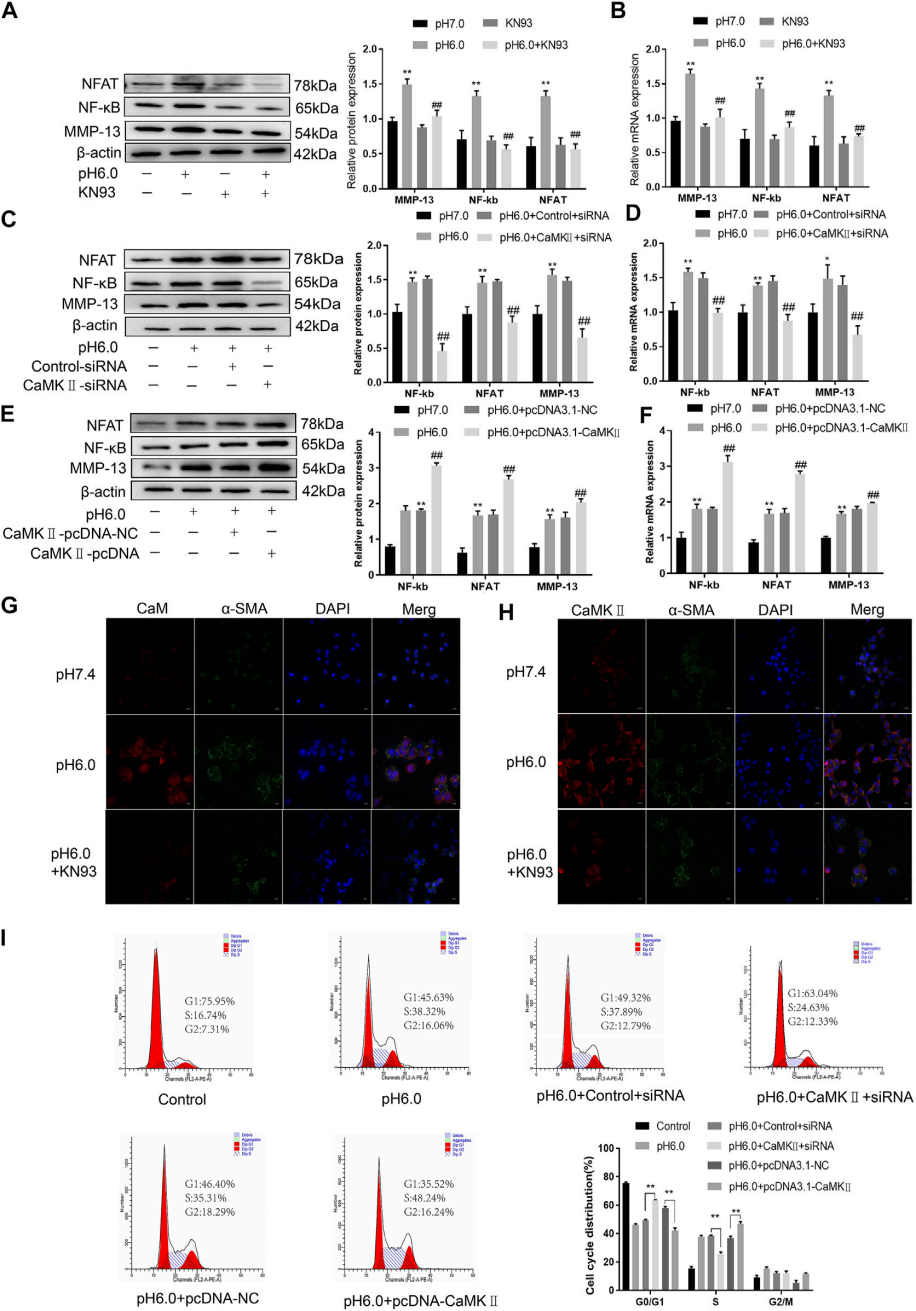
CaM/CaMKⅡ promoted the activation and proliferation of HSC. **(A)** Western blotting analysis and densitometric quantification of MMP-13, NF-kB and NFAT levels in HSCs under KN93. **(B)** mRNA levels of MMP-13, NF-kB and NFAT in HSCs treated with KN93. **(C)** Expression of MMP-13, NF-kB and NFAT in HSCs after transfection of CaMKⅡ–siRNA. **(D)** mRNA levels of MMP-13, NF-kB and NFAT in HSCs after transfection of CaMKⅡ-siRNA. **(E)** Expression of MMP-13, NF-kB and NFAT in HSCs after overexpression of CaMKⅡ. **(F)** mRNA levels of MMP-13, NF-kB and NFAT in HSCs after overexpression of CaMKⅡ. **(G,H)** The expression of CaM/CaMK Ⅱ and α-SMA was detected by immunofluorescence. (Scale Bar, 20 μM). **(I)** Flow cytometry was used to detect the effect of CaMK Ⅱ silencing or overexpression on the cell cycle of HSC-T6 cells.Data are expressed as the mean ± SD (*n* ≥ 3). *p < .05, **p < .01 vs. Control group; ^#^ *p* < .05, ^##^ *p* < .01 vs. pH 6.0 group.

These results suggest that CaM/CaMKII is expressed in HSCs of rats with HF regulated by ASIC1a, but the mechanism of CaM/CaMKII as an intracellular protein involved in the regulation of HSCs by ASIC1a in rats with HF is not clear. To this end, we first examined the effect of the CaMKII-specific inhibitor KN93 on acid-induced HSC proliferation. The cells were treated with media containing different concentrations of KN93 (1.25, 2.5, 5, 10, 20, and 40 uM). The results showed that the proliferation rate of HSCs in the pH 6.0 group was higher than that in the normal control group. Compared with the pH 6.0 group without KN93, cell proliferation decreased after adding KN93. It is concentration dependent within a certain range. It was found that KN93 (10 μM) significantly inhibited the proliferation (Figure 4A) of HSCs; therefore, this concentration was chosen as the concentration of drug action in the follow-up test. We then established a model of drug blocking, overexpression, and silencing of CaMKII. HSCs were treated with CaMKII-specific inhibitor KN93 (10 μM/ml) and then adjusted to culture medium pH 6.0 for 24 h. The results of western blotting and qRT-PCR showed that after blocking the downregulation of CaMKII by CaMKII with the specific inhibitor KN93, the expressions of CaMKII and CaM, as well as HF markers Collagen-1, *α*-SMA, and ASIC1a were decreased. Compared to the model group, the difference was statistically significant (Figures 4B, C). After CaMKII-siRNA transfection into rat HSCs, the mRNA and protein expression levels of CaMKII decreased, and the difference was statistically significant (Figures 4D, E). After specific CaMKII-siRNA transfection, the protein expression of CaMKII, CaM, and HF markers Collagen-1, *α*-SMA, and ASIC1a was downregulated. Compared to the model group, the difference was statistically significant (Figures 4F, G). The results for the CaMKII overexpression plasmid group were contrary to the above results (Figures 4H–K). The levels of protein and mRNA also changed after inhibition, silencing, and overexpression of the downstream-related proteins MMP-13, NF-κB, and NFAT (Figures 5A–F). At the same time, we detected the expression of CaM/CaMKⅡ and *α*-SMA by immunofluorescence. Compared with the normal group, the expression of CaM/CaMKII and *α*-SMA in the model group was significantly increased, while KN93 significantly inhibited the expression of CaM/CaMKII and *α*-SMA (Figures 5G, H). In addition, flow cytometry showed that acid-induced accelerated G1/S transformation was reduced after exposure to ASIC1a-siRNA, which hindered cycle progression and inhibited the proliferation of HSC-T6 cells, while overexpression of ASIC1a significantly increased the S phase and accelerated the G1/S transition of HSC-T6 cells, thus aggravating the proliferation of activated HSC-T6 cells (Figure 5I). These results suggest that the acid stimulation of ASIC1a promotes the activation and proliferation of HSC, which is related to CaM/CaMKII. In summary, CaM/CaMKII promotes HSC activation and proliferation.

### KN93 can improve the regulation of HSC activation, proliferation, and fibrosis regulated by ASIC1a

**FIGURE 6:**
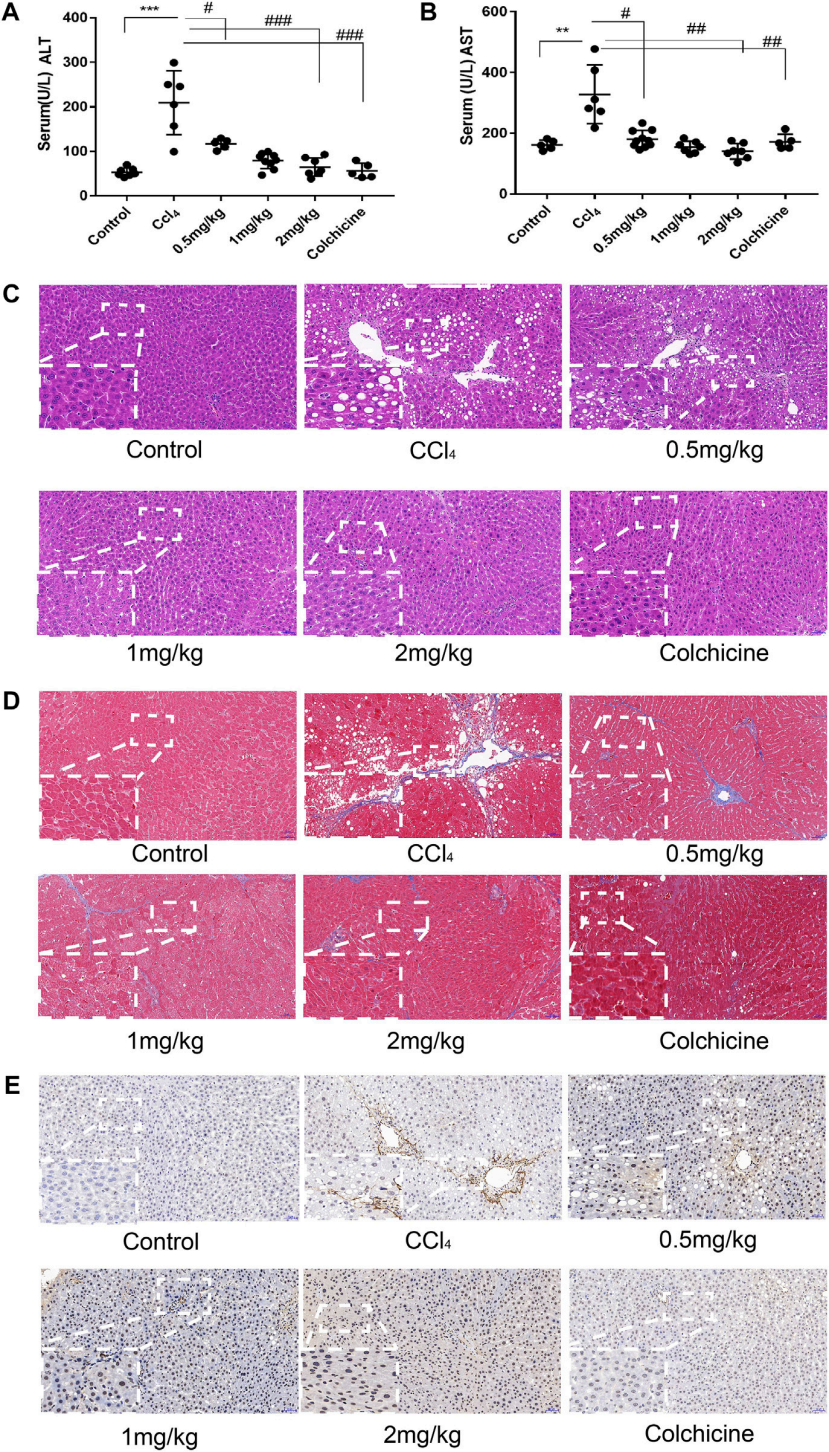
KN93 can improve ASIC1a regulation of HSC activation of proliferation and fibrosis. **(A,B)** Activities of alanine aminotransferase (ALT) and aspartate aminotransferase (AST) in the serum of rats. **(C)** Histopathological results of rat liver tissues stained with HE in each group (Scale bar, 50 µM). **(D)** Histopat hological results of rat liver tissues stained with Masson in each group (Scale bar, 50 µM). **(E)** Detection of *α*-SMA protein expression in rat liver tissue by immunohistochemical method (Scale Bar, 50 µM).

After the establishment of the model, the levels of serum alanine aminotransferase (ALT) and glutamic pyruvic transaminase (ALT) were determined graphically using GraphPadPrism7 to further verify the degree of liver injury in mice. The results showed that the level of ALT and AST in the serum of the model group was significantly higher than that of the normal group, and the difference was statistically significant. In addition, compared to the model group, KN93 (.5, 1, and 2 mg/kg) and colchicine reduced the levels of serum ALT and AST in rats, and the difference was statistically significant (*p* < .05) (Figures 6A, B). HE staining showed that the structure of the hepatic lobule was complete and clear, and the hepatocytes were arranged neatly in the normal group. In the model group, multiple false lobules were formed, steatosis and necrosis were aggravated, and vacuolation was observed in the cytoplasm. In the KN93 (1 and 2 mg/kg) and colchicine groups, the degree of fibrosis was alleviated, the hepatocytes were denatured extensively, and small red granules were observed in the cytoplasm. Compared to the model group, the degree of hepatocyte degeneration and necrosis in each treatment group was alleviated, the pathological changes were alleviated, and the cell structure was complete (Figure 6C). Masson staining showed that there was no obvious proliferation of collagen fibers in the normal group; however, the collagen fibers in the model group increased significantly and formed an unconnected fibrous septum or collagen fibers connected to the complete fibrous septum to segment the liver parenchyma. In the KN93 (1 and 2 mg/kg) and colchicine groups, the collagen fibers decreased and extended outward in a star shape from the portal area or central vein, and there was no fibrous septum formation (Figure 6D). The expression and distribution of *α*-SMA, a specific marker of activated HSCs, were analyzed using immunohistochemical techniques. The results showed that the positive expression of *α*-SMA in the liver tissue of the normal group was very low, while that of the model group increased significantly, and the positive expression area was mainly distributed in the portal vein, blood vessel wall of the hepatic portal area, and hepatic sinusoid space. The cytoplasm of the positive cells was brown, and the cell body extended. After treatment, the number of positive cells decreased significantly (Figure 6E).

### Effect of KN93 on HSC activation, proliferation, and fibrosis regulated by ASIC1a

**FIGURE 7:**
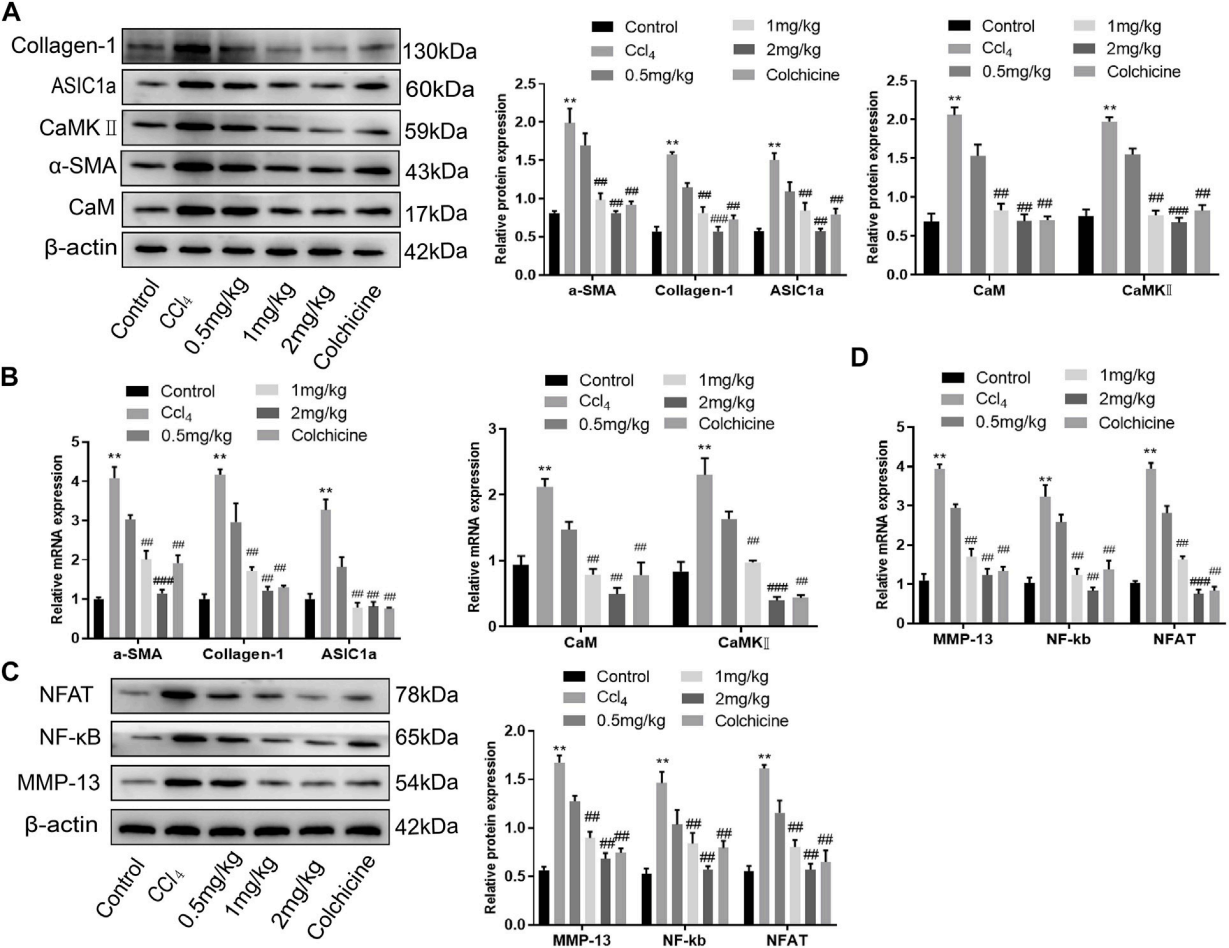
Effects of KN93 on protein and mRNA expression in liver tissue of SD rats. **(A)** Western blotting analysis and densitometric quantification of CaM, CaMKⅡ, and fibrosis proteins in HSCs of rats in each group. **(B)** mRNA levels of fibrosis protein, CaM and CaMKⅡ in HSCs of rats in each group. **(C)** Western blotting analysis and densitometric quantification of MMP-13, NF-kB and NFAT in HSCs of rats in each group. **(D)** mRNA levels of MMP-13, NF-kB and NFAT in HSCs of rats in each group. Data are expressed as the mean ± SD (*n* ≥ 3). *p < 0.05, **p < 0.01, ***p < 0.001 vs. Control group; ^#^ *p* < .05, ^##^ *p* < .01, ^###^ *p* < .001 vs. CCl_4_ group.

Western blotting showed that ASIC1a and the fibrosis markers, Collagen-1 and *α*-SMA, were highly expressed in CCl_4_-induced rat liver tissue. The expression in the model group was significantly higher than that in the normal group (*p* < .05). However, the relative expression of KN93 (1 and 2 mg/kg) and colchicine was significantly lower than that of the model group, which could reduce the expression of fibrosis marker proteins and ASIC1a. Compared with the model group, the expression of CaM and CaMKII and downstream-related proteins such as MMP-13, NF-κB, and NFAT in the KN93 (1, 2 mg/kg) and colchicine groups decreased in a dose-dependent manner (Figures 7A–C) (*p* < .05). Changes in gene expression in rats were detected using qRT-PCR. The expression levels of ASIC1a, *α*-SMA, Collagen-1, CaM, and CaMKII in the CCl_4_-induced HF group were significantly higher than those in the normal group. Compared with the model group, the expressions of fibrosis protein, ASIC1a, CaM, and CaMKⅡ in the treatment group were significantly lower than those in the model group (*p* < .05) (Figure 7B). The expression of downstream-related proteins such as MMP-13, NF-kB, and NFAT in the liver tissue of the model group was increased, while the expression of MMP-13, NF-κB, and NFAT in the liver tissue of the KN93 (1 and 2 mg/kg) and colchicine groups was significantly lower than that in the model group (*p* < .05) (Figure 7D).

## Discussion

Approximately two million people worldwide die of liver diseases every year, becoming a primary threat to human health (Asrani et al., 2019). Liver diseases have a variety of pathogenic factors, the most common being the occurrence and aggravation of viruses, alcohol consumption, and other external factors. Many chronic inflammatory diseases are accompanied by organ fibrosis, which is a common feature and an intermediate stage, accounting for 45% of all-cause mortality worldwide (Dhar et al., 2020). HF is a diffuse injury to hepatocytes accompanied by inflammation caused by various pathogenic factors. The central link to its occurrence is the excessive deposition of collagen-based extracellular matrix caused by the activation and proliferation of static HSCs (Kong et al., 2020; Lin et al., 2021). Studies have confirmed that HF is reversible in the early stages; therefore, it is extremely important to prevent or slow down the occurrence and development of hepatic fibrosis (Campana and Iredale 2017). HSC activation is closely related to HF and plays a core role in its occurrence and development (Higashi et al., 2017; Zhang et al., 2021). In the damaged liver, inhibition of the activation, proliferation, and clearance of HSCs is an important strategy for the treatment of hepatic fibrosis (Roehlen et al., 2020; Kisseleva and Brenner 2021). In this study, a model of HF was established in CCl_4_ rats. The activities of ALT and AST in the serum of rats induced by CCl_4_ increased, whereas KN93 (.5, 1, and 2 mg/kg) and colchicine decreased the activities of ALT and AST, suggesting that KN93 can reduce hepatocyte injury. HE and Masson staining showed that the treatment group could improve fibrosis. In addition, rat liver tissue was extracted, and total protein and total RNA were detected. The results showed that the expression of ASIC1a protein and liver fibrosis marker protein Collagen-1 and *α*-SMA decreased significantly after treatment. Similarly, compared with the model group, the expression of the intracellular proteins CaM and CaMKII and the downstream-related proteins MMP-13, NF-κB, and NFAT decreased. It has been suggested that KN93 can improve the regulation of HSC activation, proliferation, and fibrosis by ASIC1a.

The ASCIs is a cation channel activated by the extracellular acid of seven ASIC subunits encoded by four genes (Mango and Nistico 2020). Compared to other ASIC subunits, ASIC1a mediates extracellular Na^+^ and Ca^2+^ influx, which in turn causes a series of physiological and pathological changes in cells. (Xu et al., 2018; Wang et al., 2021b). The common pathological features of many inflammatory diseases are acidification of the local tissue environment and decreased pH (Yoder et al., 2018). Our previous studies have shown that ASIC1a is highly expressed both in rat liver fibrosis and in HSCs treated with the platelet-derived growth factor PDGF-BB. ASIC1a channels promote liver fibrosis by increasing intracellular calcium concentration. However, the specific mechanism underlying ASIC1a-induced activation and proliferation of HSCs remains unclear. In this study, western blotting and qRT-PCR were used to verify the expression of ASIC1a, fibrosis-related proteins *α*-SMA and collagen-1, and intracellular proteins CaM and CaMKII in pH 6.0-treated HSC-T6 cells. The results showed that the expression of these proteins increased after acid treatment, indicating that acid-stimulated ASIC1a promoted the activation of HSCs and that ASIC1a played a certain role in this process. PcTx-1 is a specific blocker of ASIC1a. After being blocked by specific inhibitors PcTx-1 and siRNA, the protein and mRNA expression of the HF markers *α*-SMA and collagen-1 decreased significantly, and the expression of these proteins increased after overexpression, suggesting that ASIC1a is involved in regulating the expression of *α*-SMA and collagen-1. Immunofluorescence also showed that extracellular acid promoted the expression of ASIC1a, while PcTx-1 inhibited the expression of ASIC1a. The above data suggest that acid-stimulated ASIC1a can promote the activation of HSCs and that CaM/CaMKII is expressed in HSCs of rats with HF regulated by ASIC1a.

Activation of ASIC1a induces the influx of extracellular calcium, which is an important second messenger in the cell and plays a key role in the physiological and pathological processes of the cell (Pathak and Trebak 2018). CaM is a calcium-sensitive protein that regulates the function of many proteins and plays an important role in many cellular signaling pathways. CaM can bind to different target peptides in a calcium-dependent manner, mainly by exposing hydrophobic residues (Araki et al., 2020). CaMKII is a multifunctional kinase that plays a key role in intracellular Ca^2+^ signal transduction and regulates many cellular processes, such as cell proliferation, apoptosis, gene expression, and nerve transmission (Ashraf et al., 2019; Konstantinidis et al., 2020; Chen et al., 2021). In the present study, we evaluated the potential role of CaM/CaMKII. CaMKII is a major downstream effector of Ca^2+^ signaling and plays an important role in the HF cascade (Liu et al., 2021). In this study, we observed the effect of the CaMKII-specific inhibitor KN93 on the acid-induced proliferation of HSCs. The results showed that KN93 (10 μM) significantly inhibited HSC proliferation. At the same time, the expressions of the HF markers *α*-SMA and collagen-1, intracellular proteins CaM and CaMKII, and downstream-related proteins MMP-13, NF-κB, NFAT, and mRNA were significantly decreased after blocking with the specific inhibitors of KN93 and CaMKII-siRNA. The expression of these proteins and mRNA increased after CaMKII overexpression, suggesting that CaMKII is involved in regulating the expression of these proteins. Immunofluorescence further showed that extracellular acid promoted CaMKII and *α*-SMA expression, while KN93 inhibited CaMKII and *α*-SMA expression. The expressions of ASIC1a, *α*-SMA, and collagen-1, intracellular proteins CaM and CaMKII, and downstream-related proteins MMP-13, NF-κB, and NFAT induced by CCl_4_ in rat liver tissues were significantly increased. After the injection of a specific CaMKII inhibitor, KN93, protein expression was downregulated, which was consistent with the results of the cell model. At the same time, we detected cell cycle changes in HSCs by flow cytometry. The results showed that overexpression of CaMKII promoted the G1/S phase transition and intensified the proliferation of activated HSC-T6 cells, whereas silencing CaMKII inhibited this process, resulting in a decrease in the proliferation ability of HSC-T6 cells. Our preliminary study showed that CaMKII was involved in HSC activation and proliferation. It has been suggested that CaMKII is a key signal in the regulation of the fibrotic cascade induced by HSC activation and proliferation by ASIC1a, and its blockade is a potential and effective target for the development of anti-fibrotic intervention strategies.

**FIGURE 8:**
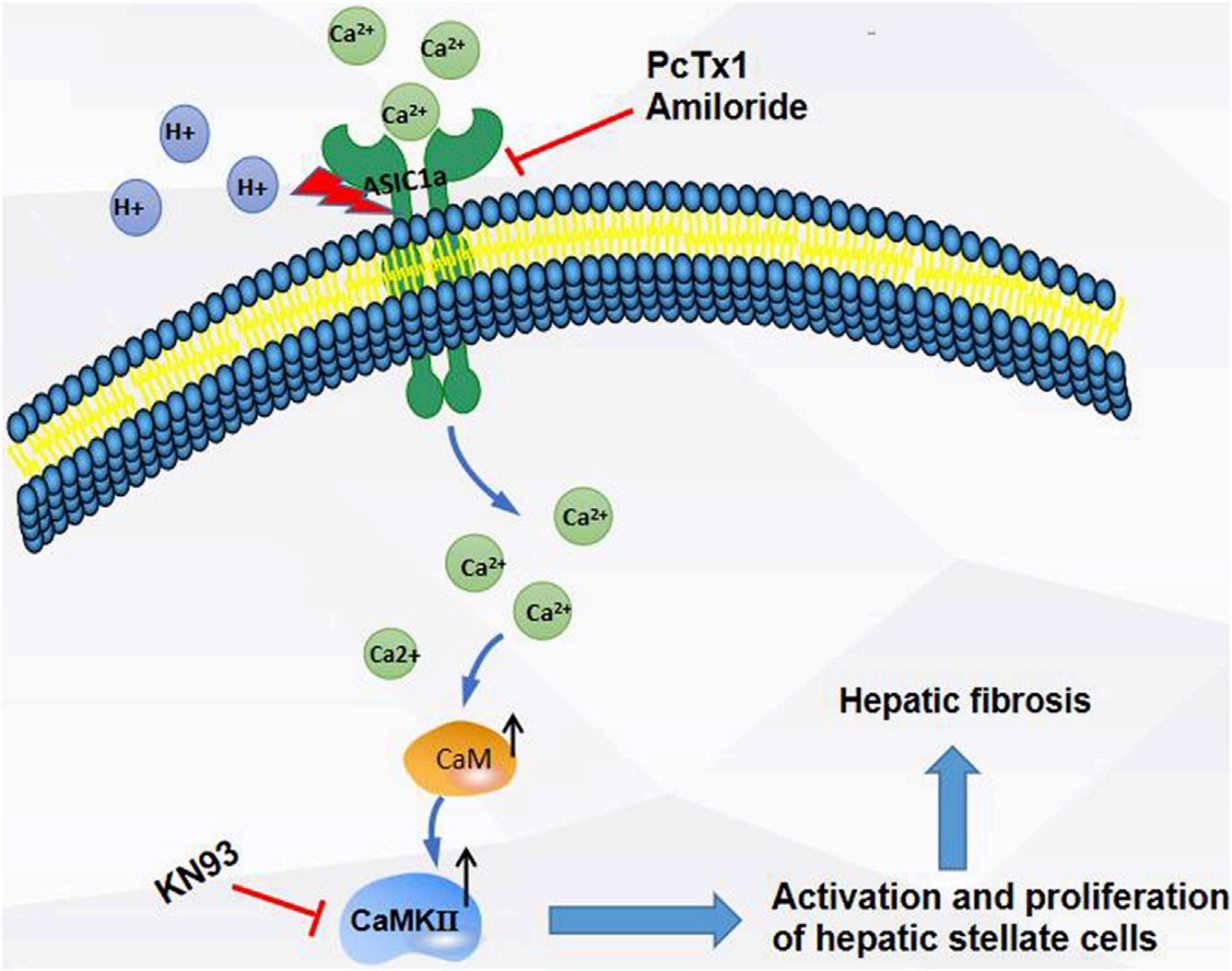
Extracellular acid activated ASIC1a channel, open channel,Ca^2+^ inflow, high expression of CaM/CaMKⅡ in HSC, activation and proliferation of HSC, suggesting that CaM/CaMKⅡ, as an intramembrane protein, participates in the regulation of HSC and hepatic fibrosis by ASIC1a.

In summary, as shown in the Figure 8, the expression of ASIC1a increased in CCl_4_-induced liver fibrosis and acid-treated HSC-T6 cells. The activation and opening of ASIC1a, the increase in the concentration of intracellular Ca^2+^, the high expression of CaM/CaMKII in HSCs, and the activation and proliferation of HSCs suggest that CaM/CaMKII is involved in the regulation of HSCs and HF by ASIC1a. However, we found that CaM/CaMKII is expressed in rat liver, which cannot fully explain the specific mechanism of CaM/CaMKII involved in ASIC1a regulation of HSC activation and proliferation. In the next step, we need to extract primary HSCs for further study and determine what specific mechanism CaMKII depends on to activate HSC proliferation. Further exploration of the specific mechanism of CaM/CaMKII in the regulation of HSC activation and proliferation by ASIC1a may provide a new strategy for the treatment of liver fibrosis.

## Data Availability

The original contributions presented in the study are included in the article/supplementary materials, further inquiries can be directed to the corresponding authors.
